# A more than four-fold sex-specific difference of autism spectrum disorders and the possible contribution of pesticide usage in China 1990–2030

**DOI:** 10.3389/fpubh.2022.945172

**Published:** 2022-09-16

**Authors:** Yang Hai, Guodong Leng

**Affiliations:** ^1^International Education College, Harbin Medical University, Harbin, China; ^2^College of Business Administration, Shenyang Pharmaceutical University, Shenyang, China

**Keywords:** autism spectrum disorders, autoregressive integrated moving average (ARIMA), Bayesian method, disability-adjusted life years (DALYs), sex-specific difference

## Abstract

Autism spectrum disorders (ASDs) are prevalent in children and adolescents and disproportionately affect males, and the main contributing factors underlying male vulnerability remain widely unknown. Pesticide use is widely reported to be associated with ASD risk, and the cases of pesticide poisoning incidence in rural areas are remarkably higher than those in the urban areas while the prevalence of ASDs in rural areas was higher than that in urban areas and the rate of male pesticide poisoning was significantly higher than female. Thus, pesticide usage may be an important contributing factor for causing sex-specific differences of ASD incidence. ASD burden was analyzed by using the data of ASD number, ASD rate (ASD cases per 100,000 persons) and disability-adjusted life years (DALYs) from 1990 to 2019. The changes from 1990 to 2030 were predicted using autoregressive integrated moving average (ARIMA) in time series forecasting based on the small values of Akaike information criterion and Bayesian information criterion. Finally, the relationship between ASD rate and pesticide usage risk index (PURI) was analyzed via Pearson's correlation coefficient. ASD number, ASD rate and DALYs will be reduced by 45.5% ± 8.2% (*t* = 9.100 and *p* = 0.0119), 56.6% ± 10.2% (*t* = 9.111 and *p* = 0.0118), and 44.9% ± 7.0% (*t* = 20.90 and *p* = 0.0023) from 1990 to 2030 in China. PURI has a strong relationship with ASD rate (rho = 0.953 to 0.988 and *p* < 0.0001). Pesticide poisoning incidence in males is up to 2-fold higher than that in females. ASD number and DALYs in males are 4-fold higher than those in females. Furthermore, there is growing evidence supporting that males are more susceptible than females to pesticides with sex differences in neurotoxicogenetics. Therefore, pesticide poisoning may be a contributing factor for causing the sex differences of ASD. Much work still needs to be done to confirm that.

## Introduction

Autism spectrum disorders (ASDs) are developmental disabilities caused by abnormalities in altered synaptic structure and function (1). ASD individuals often have strong hypersensitivity to sensory stimuli and vice versa (2). ASDs are prevalent in children and adolescents (3), and there are about one in 54 infants with ASD based on the data from CDC's Autism and Developmental Disabilities Monitoring Network (4). There are one in 59 children with autism and the rate has grown steadily for the past 20 years based on the data from the National Autism Association. ASD affected 24.8 million individuals in 2015 (5). If the future prevalence of ASD remains unchanged over the next decade, there will be an additional 1 million new cases (6). There are also conflicting prevalence estimates of ASDs in China (7). ASD disproportionately affects males (8), and the main factors contributing to male vulnerability remain widely unknown (9).

Neuropsychopathology of ASD may be the complex interplay of genetic, epigenetic, and environmental factors (10) but environmental factors may be the main trigger (9). Pesticide consumption has a strong relation with ASD (11), and will have different effects on urban and rural populations since high-levels of pesticide residue can be found in the rural soil when compared with that in urban soil (12). The cases of pesticide poisoning in rural areas are remarkably higher than those in urban areas while the prevalence of ASDs in rural areas was higher than that in urban areas (13) and the rate of males with pesticide poisoning was significantly higher than in females (14). Thus, pesticide use may be an important contributing factor for causing sex-specific differences of ASD. Early diagnosis and medical intervention for ASD will be beneficial to control autism development and reduce autism symptoms (15, 16). However, higher costs will be incurred if the effective ASD diagnosis and treatment are provided (17). Over half of children with autism live in low-income households (18). Thus, GDP per capita will affect ASD rate.

The prevalence of ASD has a seasonal epidemiological feature and shows a peak during summer (19). Some work shows the ASD rate was highest for fall births when conceived in the winter (20). Auto-regressive integrated moving average (ARIMA) is a widely used forecasting model to generalize for time series of disease analysis (21, 22). A seasonal ARIMA model will be suitable for analyzing the changing trends of ASD since its occurrence exerts seasonal characters (23, 24).

From the above information, ASDs are prevalent in children and adolescents and disproportionately affect males. Pesticide usage may be an important contributing factor for causing sex-specific differences of ASD. The probability of ASD can be evaluated by ASD number (25) and ASD rate (ASD cases per 100,000 persons) (25). Disability-adjusted life years (DALYs) are currently the most common method used for estimating the burden of disease on a population in a time interval, and ASD burden is often quantified by using DALYs (26, 27). Therefore, the potential effects of pesticide consumption on ASD risk were analyzed by using the data of ASD number, ASD rate, and DALYs from 1990 to 2019, and prediction to 2030.

## Method

### Analysis of ASD burden

ASD number (males, females, and both) represents the total ASD cases that are reported in a whole country population at a given time in China. ASD rate (males, females, and both) is calculated by using ASD cases per 100,000 persons. GBD uses DALY as a summary metric of people's health loss. The data with ASD number, ASD rate, and DALY from 1990 to 2019 were obtained from Global Burden of Disease Study 2019 (GBD, 2019) by using the tool at http://ghdx.healthdata.org/gbd-results-tool. All ASD cases were characterized by severe and pervasive impairment in social interaction and communication skills, along with restricted and repetitive patterns of behaviors or interests (28).

In 1975, the data released by the Centers for Disease Control and Prevention in USA showed that 1 in 5,000 children had an ASD, and by 2014, one in 68 children had an ASD. The autism rate shows an upward trend year by year, which is related to the change of diagnostic standards, the improvement of public awareness, and the improvement of diagnosis level (29). Higher rates of GDP growth per capita were found to be associated with a deterioration of mental health in China (30). Socioeconomic status is closely associated with cases of autism diagnosis (31). Thus, GDP per capita will affect ASD rate. Real GDP growth was adjusted by GDP deflator and increased by 3.48 fold from 2004 to 2018, which is subjective to linear regression (R^2^ = 0.9957) (32). The productivity of capital were relatively high in the eastern (financial efficiency evaluation index (FEEI), 0.27 ± 0.08) and central regions (FEEI, 0.27 ± 0.10), while they were relatively low in the western regions (FEEI, 0.24 ± 0.07) (33). On the other hand, the ASD rate was 28.7 per 10,000 in 2004 (34) and a mainstream ASD rate was 39.23 per 10,000 in 2018 (34), and increased by 1.37 fold from 2004 to 2018. ASD cases were negatively adjusted by the fold changes of real GDP growth since socioeconomic status is closely associated with cases of autism diagnosis and normalized with the ASD rate change from 2004 to 2018.

A seasonal ARIMA model was used to analyze the data with ASD number, ASD rate, and DALY (from 1990 to 2019) in China and compares the quarter-on-quarter changes after the seasonal adjustment (22.9–23.9%, 23.1–24.7%, 26.5–27.7%, and 24.3–26.3% for spring, summer, fall, and winter, respectively) according to what was previously reported (20, 35). ASD rate was highest for fall births and lowest for spring births (20, 35) and the autism rate in China is comparable to Western prevalence (36).

A seasonal ARIMA (p,d,q) (P,D,Q) model comprises the following components:

1) The Auto-Regressive (AR) part

$$\begin{array}{l} y_{t}=\mu +\overset{p}{\underset{i=1}{\sum }}\gamma_{i}y_{t-i}+\epsilon_{t} \end{array}$$

2) The Moving Average (MA) part

$$\begin{array}{l} y_{t}=\mu +\overset{q}{\underset{i=1}{\sum }}\theta_{i}\epsilon_{t-i}+\epsilon_{t} \end{array}$$

3) ARIMA part

$$\begin{array}{l} y_{t}=\mu +\overset{p}{\underset{i=1}{\sum }}\gamma_{i}y_{t-i}+\epsilon_{t}+\overset{q}{\underset{i=1}{\sum }}\theta_{i}\epsilon_{t-i} \end{array}$$

y denotes the series of observations, γ and θ present the parameters for the AR and MA parts of the model respectively, ϵ stands for the prediction errors, and μ is the intercept parameter. P and Q are the numbers of periods included in the seasonal AR and MA parts of the model, and D is the number of seasonal differences.
AR and MA orders were identified by Autocorrelation function (ACF), and Partial autocorrelation function (PACF) plots.
The model was chosen according to the lowest values of Akaike information criterion (AIC) and Bayesian information criterion (BIC) as follows. Time Series ARIMA model fits AIC -pdq/s by grid search.

$$\begin{array}{lll} \text{AIC} & = & 2\text{K}-2\text{ln}\left(\text{L}\right)\\ \text{BIC} & = & \text{Kln}\left(\text{n}\right)-2\text{ln}\left(\text{L}\right) \end{array}$$

K presents the number of estimated parameters in the model, *L* equals the maximized likelihood function, and n stands for the number of samples.
  ASD burden was analyzed using the above seasonal ARIMA model and predicted to 2030.

### The changes of ASD burden from 1990 to 2030

Based on the data from GBD 2019 and predicted data using the ARIMA model, the changes of ASD number, ASD rate, and DALYs from 1990 to 2030 were compared by using *t*-student at 10-year intervals. ASD numbers, ASD rates, and DALYs between the range (the lower and the upper limit) were provided for a specific period of time. The paired *t*-test was used to test whether there were significant differences between different years by using GraphPad Prism 8 (California, USA). *P* < 0.05 is considered significant for the differences.

### The relationship between pesticide usage risk index (PURI) and ASD rate

Rural areas are known as the most exposed pesticide due to plantation fields. Given that pesticide usage is quite different in urban areas as opposed to rural areas, the changes of urban and rural population will affect pesticide exposure. Rural-urban ratio of pesticide poisoning rate was about 3.5 to 1 (as a supposed risk coefficient) in China (37, 38). Here, we introduced the concept of PURI to stand for the relative risk degrees of using pesticide. PURI = pesticide usage (kg/person) × (rural/urban population ratio) × 3.5. Therefore, PURI was adjusted *via* pesticide usage (Supplementary Figure S1), and China population and changes of urban and rural population (Supplementary Figure S2). Pesticide usage has striking seasonal variability. Using the above method, pesticide consumption (from 1990 to 2021) in China and compares the quarter-on-quarter changes after the seasonal adjustment (12.3–30.7%, 30.7–49.2%, 15.4–30.7%, and 15.4–17.4% for spring, summer, fall, and winter, respectively) with peak pesticide concentrations occurring in summer according to previously reported data (39–41). Pesticide use was analyzed using the above seasonal ARIMA model and predicted to 2030. The relationship between PURI and ASD rate was evaluated by using the Pearson Correlation Coefficient Calculator.

## Results

### The changes of ASD number and rate from 1990 to 2030

**Figure 1:**
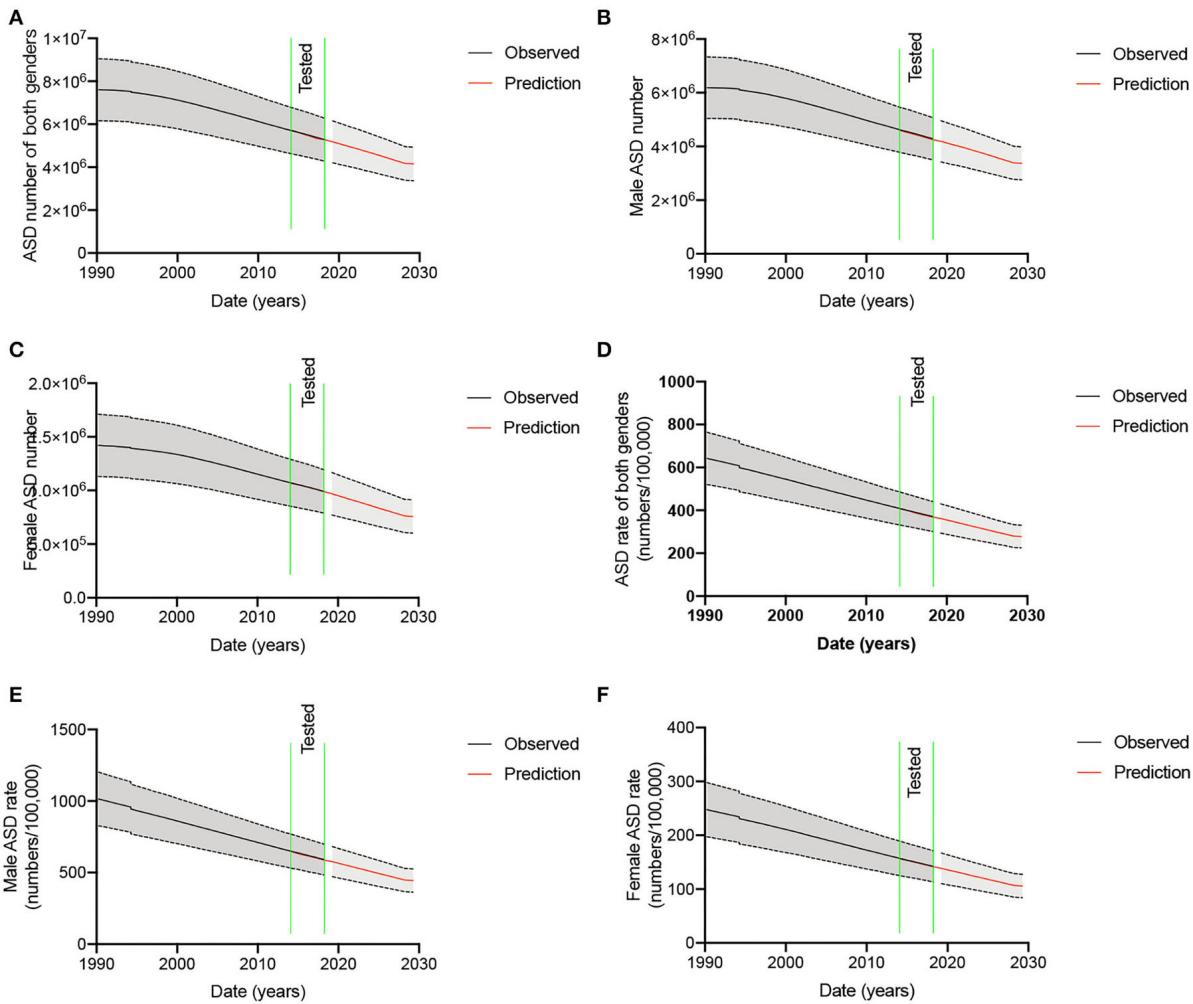
ASD number and ASD rate in the mainland of China, 1990-2030. **(A)** ASD number of both genders. **(B)** ASD number of males. **(C)** ASD number of females. **(D)** ASD rate of both genders. **(E)** ASD rate of males. **(F)** ASD rate of females.

The seasonal ARIMA model shows superior forecasting ability that the observed data are consisted with the tested data between 2015 and 2019 (Figure 1A). ASD numbers of both genders will be reduced by 45.5% ± 8.2% (*t* = 9.100 and *p* = 0.0119) in China, 1990–2030. ASD number of males will be reduced by 44.6% ± 8.0% (*t* = 9.258 and *p* = 0.0115) from 1990 to 2030 in China (Figure 1B). ASD number of females will be reduced by 46.7% ± 8.4% (*t* = 8.497 and *p* = 0.0136) from 1990 to 2030 in China (Figure 1C).

Similarly, the ASD rates in both genders have been reducing from 1990 to 2019. The seasonal ARIMA model shows superior forecasting ability that the observed data are consistent with the tested data between 2015 and 2019 (Figure 1D). The ASD rate of both genders will be reduced from 642.3 ± 121.9 to 277.8 ± 52.6 per 100,000 persons (56.6% ± 10.2%, *t* = 9.111 and *p* = 0.0118, Figure 1D) from 1990 to 2030 in China. Similarly, the rates were reduced from 1990 to 2019 in males. The seasonal ARIMA model shows superior forecasting ability that the observed data are consistent with the tested data between 2015 and 2019 in males (Figure 1E). The ASD rate of males will be reduced from 1014.0 ± 187.6 to 444.6 ± 81.5 per 100,000 persons (56.2% ± 10.1%, *t* = 9.294 and *p* = 0.0114, Figure 1E) from 1990 to 2030 in China. The rates were also reducing consistently from 1990 to 2019 in females. The seasonal ARIMA model shows superior forecasting ability that the observed data are consistent with the tested data between 2015 and 2019 in females (Figure 1F). The ASD rate of females will be reduced from 247.7 ±50.6 to 105.8±21.7 per 100,000 persons (57.3% ± 10.3%, *t* = 8.491 and *p* = 0.0136, Figure 1F) from 1990 to 2030 in China. The numbers and ASD rates of males are more than 4-fold than those in females (Figures 1B,C,E,F).

**Figure 2:**
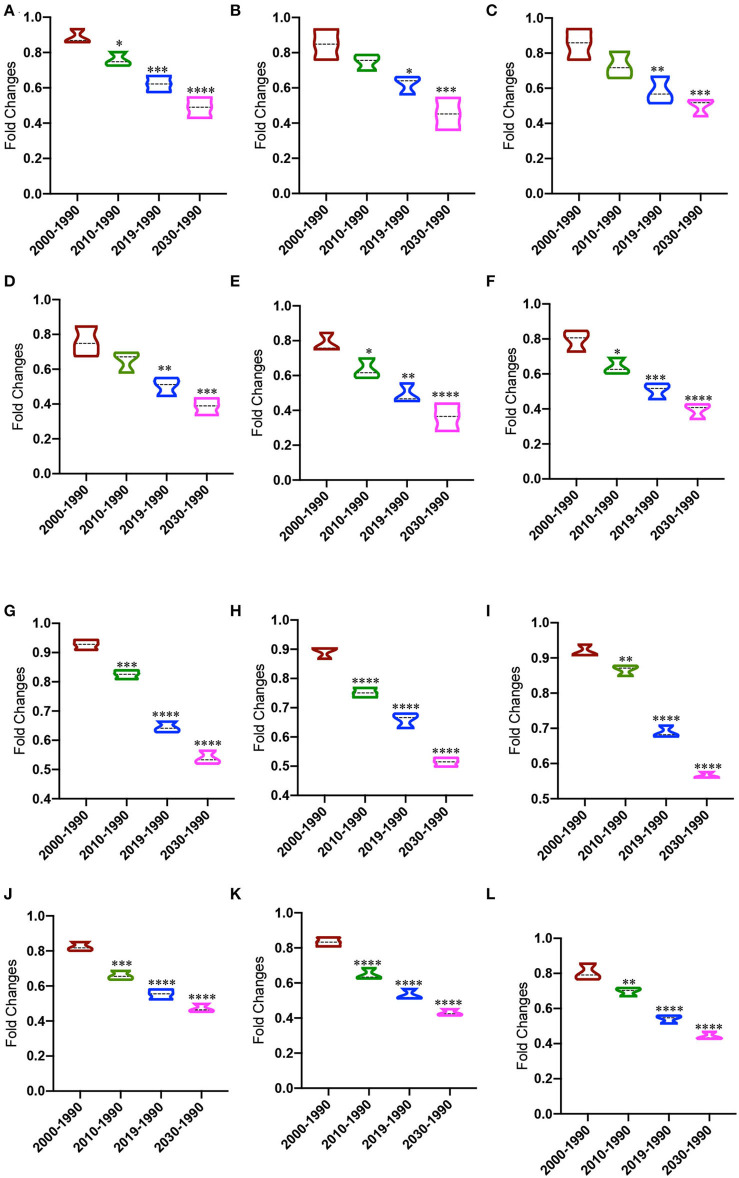
The changing trend of ASD and DALYs from 1990 to 2030. **(A)** The fold changes of ASD number of both genders. **(B)** The fold changes of ASD number of males. **(C)** The fold changes of ASD number of females. **(D)** The fold changes of ASD rate in both genders. **(E)** The fold changes of ASD rate in males. **(F)** The fold changes of ASD rate in females. **(G)** The fold changes of DALYs number in both genders. **(H)** The fold changes of DALYs number in males. **(I)** The fold changes of DALYs number in females. **(J)** The fold changes of DALYs rate in both genders. **(K)** The fold changes of DALYs rate in males. **(L)** The fold changes of DALYs rate in females. *P < 0.05, **P < 0.01, ***P < 0.001, and ****P < 0.0001 vs. 2000–1990 group.

The fold changes for ASD numbers of both genders were reduced from 1990 to 2010, 2019, and 2030 when compared with those from 1990 to 2000 (Figure 2A, *p* = 0.025, *p* = 0.0003, and *p* < 0.0001, respectively). The fold changes for ASD number of males were reduced from 1990 to 2019 and 2030 when compared with those from 1990 to 2000 (Figure 2B, *p* = 0.012, and *p* = 0.0004, respectively). The fold changes for ASD number of females were reduced from 1990 to 2019 and 2030 when compared with those from 1990 to 2000 (Figure 2C, *p* = 0.005 and *p* = 0.0009, respectively). The fold changes for ASD rates of both genders were reduced from 1990 to 2010, 2019, and 2030 when compared with those from 1990 to 2000 (Figure 2D, *p* = 0.048 *p* = 0.0014 and *p* = 0.0001, respectively). The fold changes for ASD rate of males were reduced from 1990 to 2010, 2019, and 2030 when compared with those from 1990 to 2000 (Figure 2E, *p* = 0.042, *p* = 0.0011 and *p* < 0.0001, respectively). The fold changes for ASD rates of females were reduced from 1990 to 2010, 2019, and 2030 when compared with those from 1990 to 2000 (Figure 2F, *p* = 0.014, *p* = 0.0003 and *p* < 0.0001, respectively). The fold changes of ASD rates were significantly lower than those of ASD numbers possibly because of increased China population.

### The changes of ASD-caused DALYs from 1990 to 2030

The fold changes for ASD-caused DALYs number of both genders were reduced from 1990 to 2010, 2019, and 2030 when compared with those from 1990 to 2000 (Figure 2G, *p* = 0.0004, *p* < 0.0001 and *p* < 0.0001, respectively). The fold changes for DALYs number of males were reduced from 1990 to 2010, 2019, and 2030 when compared with those from 1990 to 2000 (Figure 2H, *p* < 0.0001, *p* < 0.0001 and *p* < 0.0001, respectively). The fold changes for DALYs number of females were reduced from 1990 to 2010, 2019, and 2030 when compared with those from 1990 to 2000 (Figure 2I, *p* = 0.006, *p* < 0.0001 and *p* < 0.0001, respectively). The fold changes for DALYs rate of both genders were reduced from 1990 to 2010, 2019, and 2030 when compared with those from 1990 to 2000 (Figure 2J, *p* = 0.0002, *p* < 0.0001 and *p* < 0.0001, respectively). The fold changes for DALYs rate of males were reduced from 1990 to 2010, 2019, and 2030 when compared with those from 1990 to 2000 (Figure 2K, *p* < 0.0001, *p* < 0.0001 and *p* < 0.0001, respectively). The fold changes for DALYs rate of females were reduced from 1990 to 2010, 2019, and 2030 when compared with those from 1990 to 2000 (Figure 2L, *p* = 0.007, *p* < 0.0001 and *p* < 0.0001, respectively).

### PURI has a strong relationship with ASD rate

**Figure 3:**
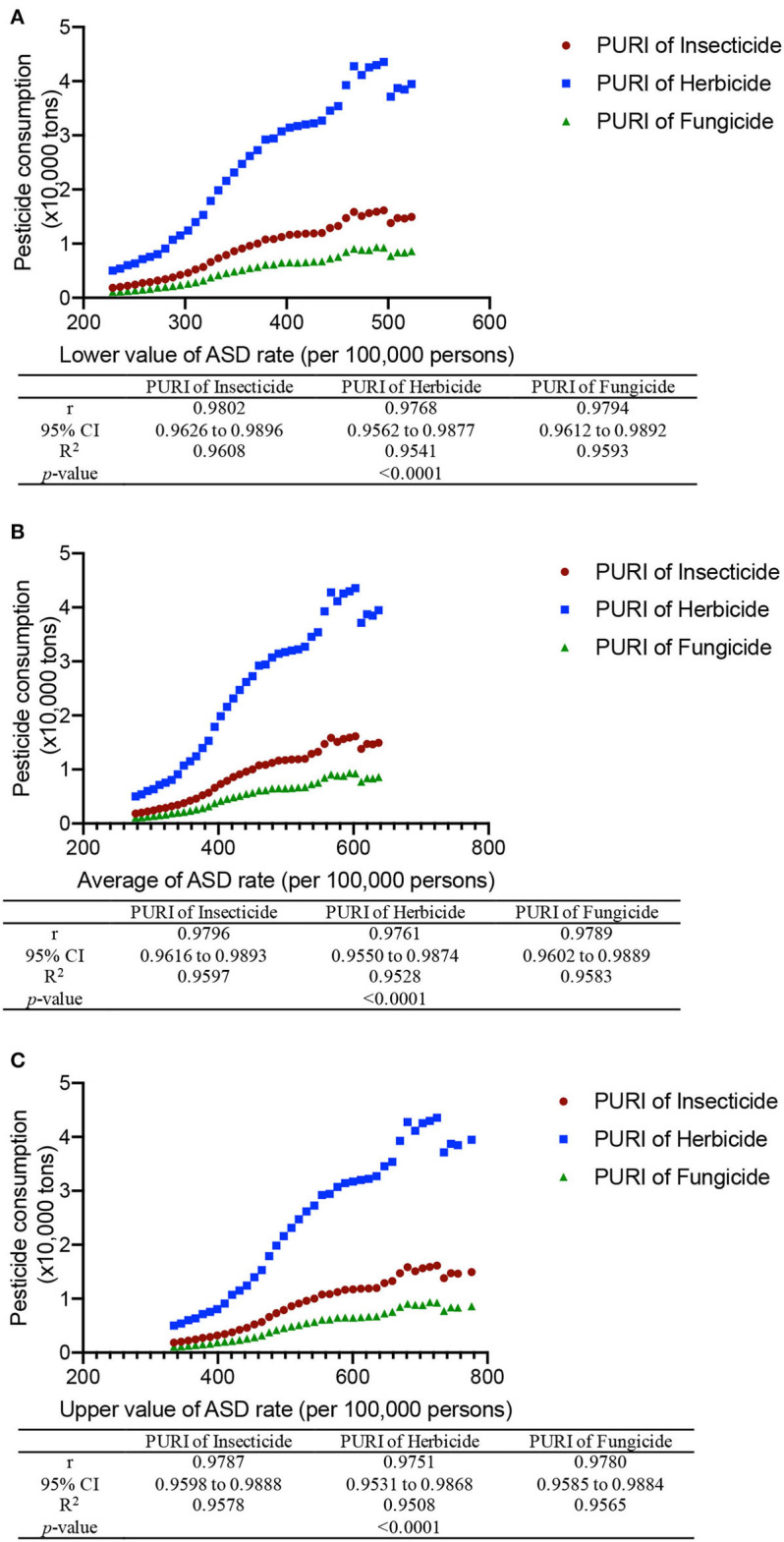
Pearson's correlation analysis of the relationship between ASD rate and pesticide usage risk index (PURI) in China from 1990 to 2030. **(A)** The relationship between the lower limitation of ASD rate and PURI. **(B)** The relationship between the average of ASD rate and PURI. **(C)** The relationship between the upper limitation of ASD rate and PURI. Pesticides include fungicide, herbicide, and insecticide. PURI = pesticide usage (kg/person) × (rural/urban population ratio) × 3.5. Spearman's Rho is a non-parametric test to evaluate the strength of association between the two variables, where r = 1 means a perfect positive correlation.

China pesticide consumption has been significantly reduced since 2015 and will be reduced < 1,000,000 tons by 2030 (Supplementary Figure S1). China's population will reach 14.6 × 10^8^ while urban population will be near 3-fold of rural population (Supplementary Figure S2). Thus, PURI was calculated according to these data at the specific date by using the equation, PURI = pesticide usage (kg/person) × (rural/urban population ratio) × 3.5. The results show that PURI has a strong relationship with the upper, lower, and average values of the ASD rate (Figures 3A–C, rho = 0.953 to 0.988 and *p* < 0.0001), suggesting that pesticide usage may be an important contributing factor for causing ASD risk.

### Pesticide poisoning possibly causes the sex differences of ASD

Pesticide poisoning incidence in males is higher than that in females in most places in China, and the incidence in males is near 2-fold higher than that in the females (Supplementary Table S1). Furthermore, there is growing evidence showing that males are more susceptible than females to the adverse effects of pesticides with sex differences in neurotoxicogenetics (42). Therefore, pesticide poisoning may be a contributing factor for causing the sex differences of ASD since ambient pesticide exposure will increase ASD risk (43).

## Discussion

In this study, we estimated and projected the trends in ASD number, ASD rate, and ASD-caused DALYs in males, females, and both genders in China during 1990-2030. ASD numbers and ASD rate are consistently reducing and there will be a significant difference from 1990 to 2030 (Figure 1). ASD constituted the principal number of attributable DALYs in China while the estimated prevalence of ASD in China has been consistently reported to be lower than that in the West (44). ASD accounts for the greater burden compared with other mental disorders (for instance, schizophrenia and bipolar disorder), which consumes most mental health resources (45, 46). Changes in epidemiological parameters, primarily prevalence, will also have major effects on future trends.

ASD number, ASD rate, and ASD-caused DALYs in males are 4-fold more than those in females, and pesticide may be a risk for causing autism incidence (11, 47). The pesticide poisoning cases in rural areas is near 2-fold more than those in urban areas (13). Theoretically, with the rural-urban migration and reduction in chemical pesticide usage, it is quite reasonable to propose that ASD rates will be reduced since ASD risk has a close relationship with pesticide consumption (11) and its pollution in the rural areas is significantly higher than that in urban areas (48). Pesticide poisoning incidence in males is higher than that in females in most places in China, and the incidence in males is near 2-fold higher than that in the females (Supplementary Table S2). Sex-specific difference of ASD may be mainly caused by more chance of pesticide poisoning in males.

Important limitations in GBD methods should be considered when interpreting these estimates. First, an approach is used to calculate bounds of uncertainty in GBD from 1990 to 2019, which will envelop all possible results; it is possible that uncertainty intervals around the 2030 DALY estimates are overestimated because of unknown effects of the present methods, or are wider, as a result. Conversely, the modeling software used to derive uncertainty in burden projections (Ersatz) is unable to capture all sources of uncertainty in these calculations and therefore produces uncertainty intervals that might underestimate true uncertainty. Country-level burden estimates do not capture effects with a local context (49). Ecological differences and other culture-specific variables were not comprehensively captured in GBD from 1990 to 2019 and this is of particular relevance in a large country like China where regional differences and environmental factors could be associated with variations in prevalence rates for some ASDs (50, 51).

According to the reported literatures, pesticide poisoning incidence in males is higher than that in females in China by more than 2-fold (Supplementary Table S1). Furthermore, there is much evidence supporting that males are more susceptible than females to pesticides usages and that only males are affected by some neurotoxicants, which may be caused by sex differences in neurotoxicogenetics (42, 52). Therefore, pesticide poisoning may be a potential contributing factor for causing the sex disparities of ASD since ambient pesticide exposure will increase ASD risk (43). Unfortunately, we still have not found the strong effects of pesticide usage on the sex-specific difference of ASD yet with the change of urban/rural populations. Other potential confounders were not well justified. For instance, rural-urban migration may cause very high sex ratio of males in some rural areas in China, and the ratio of unmarried males to females can reach 1.9 (53). In some rural counties, the ratio approaches 130:100 (54). Furthermore, the reduction rate of chemical pesticide usage is significantly slower than the reduction of rural population, suggesting that each person may contact much more chemical pesticide than before. To address these important issues, much work needs to be done to ensure the exact effects of chemical pesticide usage.

China achieved the zero growth of chemical pesticide consumption since 2015, which is earlier than the schedule of 2020 (55). The remarkable reduction in chemical pesticide consumption is closely associated with agricultural safety policy improvement in China (56). The Chinese government needs to further strengthen pesticide control and improve the diagnosis and therapy of ASD and health insurance. On the other hand, to maintain high-level crop yields, China needs to explore and develop biological pesticides to replace chemical pesticides to keep the normal life of the largest population in China. Meanwhile, China has great challenges to control pesticide pollution and improve the environment.

## Data availability statement

The original contributions presented in the study are included in the article/Supplementary material, further inquiries can be directed to the corresponding author.

## Ethics statement

Ethical review and approval was not required for the study on human participants in accordance with the local legislation and institutional requirements. Written informed consent from the patients/participants or patients/participants' legal guardian/next of kin was not required to participate in this study in accordance with the national legislation and the institutional requirements.

## Author contributions

YH and GL designed the experiment, collected, analyzed, and analyzed all data. GL wrote the paper. YH revised the paper. Both authors agreed with the final submission. Both authors contributed to the article and approved the submitted version.

## Conflict of interest

The authors declare that the research was conducted in the absence of any commercial or financial relationships that could be construed as a potential conflict of interest.

## Publisher's note

All claims expressed in this article are solely those of the authors and do not necessarily represent those of their affiliated organizations, or those of the publisher, the editors and the reviewers. Any product that may be evaluated in this article, or claim that may be made by its manufacturer, is not guaranteed or endorsed by the publisher.

## Supplementary material

Supplementary Table S1 Pesticide poisoning cases in child in China.

Supplementary Figure S1 Chemical pesticide consumption in China from 1991 to 2030. Pesticides include fungicide, herbicide and insecticide.

Supplementary Figure S2 The changes of urban and rural population from 1990 to 2030 in China.
