# Supplementary material for: A more than four-fold sex-specific difference of autism spectrum disorders and the possible contribution of pesticide usage in China 1990–2030
Source: Front Public Health. 2022 Sep 16;10:945172. doi: 10.3389/fpubh.2022.945172 (PMC9525129; doi:10.3389/fpubh.2022.945172)
Supplement: Supplementary Table S1 — Pesticide poisoning cases in child in China. [file Table_1.DOCX]

Table S1. Pesticide poisoning cases in child in China

| Date | Regions | Males | | Female | | References |
| --- | --- | --- | --- | --- | --- | --- |
|  |  | Cases | Age | Cases | Age |  |
| Feb, 2013 | Fugou, Henan | 51 (57.3%) | 10 m- 12 y | 38(42.7%) | 10 m- 12 y | [1] |
| Jan, 2000-Nov, 2009 | Fengcheng, Jiangxi | 64 (54.7%) | 3 m- 14 y | 53(45.3%) | 3 m- 14 y | [2] |
| 2008 | Shanghai | 162 (53.8%) | 3-10 y (76.7%) | 139 (46.2%) | 3-10 y (76.7%) | [3] |
| Jan. 2012 to Dec. 2020 | Chongqing | 588 (54.6%) | Pre-adolescence (85.4%) | 488 (45.4%) | Pre-adolescence (85.4%) | [4] |
| Sep.2018 to Jan.2019 | Wuhan | 40 (58.0%) | 1-3.3 y | 29 (42.0%) | 1-3.3 y | [5] |
| Sep.2018 to Jan.2019 | Shenzhen | 43 (61.4%) | 1.8-5 y | 27 (36.6%) | 1.8-5 y | [5] |
| June 2016 to July 2017 | Shanghai | 172 (56.8%) | 7.34 ± 0.35 | 131 (43.2%) | 7.34 ± 0.35 | [6] |
| 2014 to 2015 | Taipei, Taiwan | 126 (69.6) | 8.9 ± 2.4 | 55 (30.4) | 8.9 ± 2.4 | [7] |
| June 2011–Jan. 2012 | Jiangsu | 205(56.3%) |  | 159(43.7%) |  | [8] |
| 2020 | Wuhan | 544 (52.4%) | 6-24 m | 495 (47.6%) | 6-24 m | [9] |
| Sep. 2010 to Dec. 2013 | Shanghai | 397 (51.4%) | 357 (46.2%) <9y | 376 (48.6%) | 357 (46.2%) <9y | [10] |
| 2007-2018 | Bengbu, Anhui | 1208(68.4%) | 0-14y | 687 (31.6) | 0-14y | [11] |
| 2012-2018 | Ya’an, Sichuan | 73(66.4%) | Before school | 37(33.6%) | Before school | [12] |
| Jan-Dec, 2013 | Chuzhou, Zhejiang | 43(55.1%) | 7.06±3.25 | 35 (44.9%) | 7. 13 ± 3. 21 | [13] |

**References**

1. Zou A, Li H. Clinical analysis of 89 children with organophosphorus pesticide poisoning in primary hospitals. Henan Medical Research. 2014;23(3):49-51.

2. Fan M, Lei Z. Retrospective analysis of 117 children with pesticide poisoning. Modern Preventive Medicine. 2011;38(9):1639-40.

3. Guodong D, Pei W, Ying T, Jun Z, Yu G, Xiaojin W, et al. Organophosphate pesticide exposure and neurodevelopment in young Shanghai children. Environmental science & technology. 2012;46(5):2911-7.

4. Zhao Y, Zhu Z, Xiao Q, Li Z, Jia X, Hu W, et al. Urinary neonicotinoid insecticides in children from South China: Concentrations, profiles and influencing factors. Chemosphere. 2022;291:132937.

5. Song W, Wan Y, Jiang Y, Liu Z, Wang Q. Urinary concentrations of 2, 4-D in repeated samples from 0–7 year old healthy children in central and south China. Chemosphere. 2021;267:129225.

6. Zhang J, Guo J, Wu C, Qi X, Jiang S, Zhou T, et al. Early-life carbamate exposure and intelligence quotient of seven-year-old children. Environment International. 2020;145:106105.

7. Chang C-H, Yu C-J, Du J-C, Chiou H-C, Hou J-W, Yang W, et al. The associations among organophosphate pesticide exposure, oxidative stress, and genetic polymorphisms of paraoxonases in children with attention deficit/hyperactivity disorder. Science of the Total Environment. 2021;773:145604.

8. Liu P, Wu CH, Chang XL, Qi XJ, Zheng ML, Zhou ZJ. Assessment of chlorpyrifos exposure and absorbed daily doses among infants living in an agricultural area of the Province of Jiangsu, China. Int Arch Occup Environ Health. 2014;87(7):753-62. PMID: 24257932. doi: 10.1007/s00420-013-0918-1.

9. Yang C, Fang J, Sun X, Zhang W, Li J, Chen X, et al. Prenatal exposure to organochlorine pesticides and infant growth: A longitudinal study. Environment International. 2021;148:106374.

10. Pan C, Yu J, Yao Q, Lin N, Lu Z, Zhang Y, et al. Prenatal neonicotinoid insecticides Exposure, oxidative Stress, and birth outcomes. Environment International. 2022;163:107180.

11. Zhu F, Wang C, Qian Q. Epidemiological analysis of pesticide poisoning in children aged 0-14 years in Bengbu City from 2007 to 2018. Chinese School Health. 2019;8.

12. Li M, Feng D, Liu N. Analysis of pesticide poisoning among children and adolescents in Ya'an from 2012 to 2018. Journal Preventive Medicine. 2021;37(9):1264-8.

13. Fan L. Analysis of risk factors of pesticide poisoning in 78 rural children. China Maternal and Child Health. 2014;29(36):6049-50.

**Figure legends**

Figure S1. Chemical pesticide consumption in China from 1991 to 2030. Pesticides include fungicide, herbicide and insecticide.

Figure S2. The changes of urban and rural population from 1990 to 2030 in China.

Figure S1

Figure S2
